# Evaluating the effects of continuous veno-venous hemodiafiltration on O_2_ and CO_2_ removal and energy expenditure measurement using indirect calorimetry

**DOI:** 10.1186/s13613-025-01426-2

**Published:** 2025-01-11

**Authors:** Weronika Wasyluk, Robert Fiut, Marcin Czop, Agnieszka Zwolak, Wojciech Dąbrowski, Manu L N G Malbrain, Joop Jonckheer

**Affiliations:** 1 Department of Internal Medicine and Internal Medicine in Nursing, Medical University of Lublin, Lublin, Poland; 2 Department of Clinical Physiotherapy, Medical University of Lublin, Lublin, Poland; 3 Department of Clinical Genetics, Medical University of Lublin, Lublin, Poland; 4 1st Department of Anaesthesiology and Intensive Therapy, Medical University of Lublin, Lublin, Poland; 5 Medical Data Management, Medaman, Geel, Belgium; 6 International Fluid Academy, Lovenjoel, Belgium; 7 Department of Intensive Care Medicine, Universitaire Ziekenhuis Brussel (UZ Brussel), Vrije Universiteit Brussel (VUB), Brussels, Belgium

**Keywords:** Oxygen, Carbon dioxide, Indirect calorimetry, Resting energy expenditure, Respiratory quotient, Sepsis, Continuous renal replacement therapy, CRRT

## Abstract

**Background:**

Continuous veno-venous hemodiafiltration (CVVHDF) is used in critically ill patients, but its impact on O₂ and CO₂ removal, as well as the accuracy of resting energy expenditure (REE) measurement using indirect calorimetry (IC) remains unclear. This study aims to evaluate the effects of CVVHDF on O₂ and CO₂ removal and the accuracy of REE measurement using IC in patients undergoing continuous renal replacement therapy.

**Design:**

Prospective, observational, single-center study.

**Methodology:**

Patients with sepsis undergoing CVVHDF had CO₂ flow (QCO₂) and O₂ flow (QO₂) measured at multiple sampling points before and after the filter. REE was calculated using the Weir equation based on V̇CO₂ and V̇O₂ measured by IC, using true V̇CO₂ accounting for the CRRT balance, and estimated using the Harris-Benedict equation. The respiratory quotient (RQ), the ratio of V̇CO₂ to V̇O₂, was evaluated by comparing measured and true values.

**Results:**

The mean QCO₂ levels measured upstream of the filter were 76.26 ± 17.33 ml/min and significantly decreased to 62.12 ± 13.64 ml/min downstream of the filter (*p* < 0.0001). The mean QO₂ levels remained relatively unchanged. The mean true REE was 1774.28 ± 438.20 kcal/day, significantly different from both the measured REE of 1758.59 ± 434.06 kcal/day (*p* = 0.0029) and the estimated REE of 1619.36 ± 295.46 kcal/day (*p* = 0.0475). The mean measured RQ value was 0.693 ± 0.118, while the mean true RQ value was 0.731 ± 0.121, with a significant difference (*p* < 0.0001).

**Conclusions:**

CVVHDF may significantly alter QCO₂ levels without affecting QO₂, influencing the REE and RQ results measured by IC. However, the impact on REE is not clinically significant, and the REE value obtained via IC is closer to the true REE than that estimated using the Harris-Benedict equation. Further studies are recommended to confirm these findings.

**Graphical Abstract:**

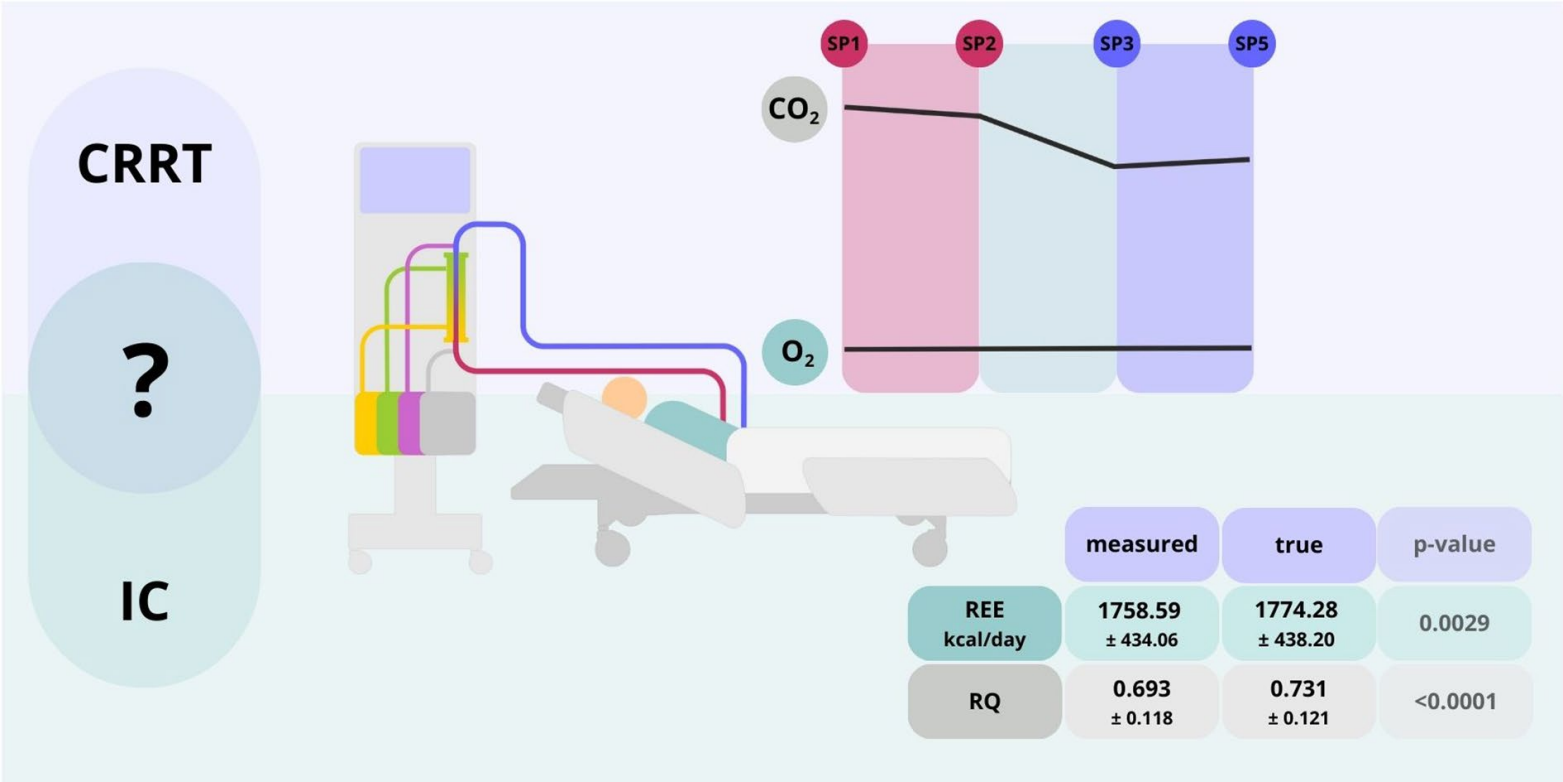

## Introduction

Sepsis is defined as *a life-threatening organ dysfunction caused by a dysregulated host response to infection* [1]. Dysregulated host response includes, among others, changes in basic metabolic processes [2]. Indirect calorimetry (IC) is the recommended method for assessing energy expenditure (EE) in critically ill mechanically ventilated patients. The weakness of predictive equations and the use of IC have been subject to multiple evaluations and recommendations from international nutrition societies, such as European Society for Clinical Nutrition and Metabolism (ESPEN), preferring the use of indirect calorimetry to evaluate the actual energy expenditure of ICU patients [3]. The determination of resting energy expenditure (REE) using the indirect calorimetry (IC) method requires measuring the fraction of inspired oxygen (FiO_2_), the fraction of expired oxygen (FeO_2_), the fraction of expired carbon dioxide (FeCO_2_), and the minute volume of expired gas. These parameters allow for calculating oxygen consumption (V̇O_2_) and carbon dioxide production (V̇CO_2_). Subsequently, these values are used to calculate REE via the Weir equation. [4, 5]. Another parameter that can be calculated based on V̇O_2_ and V̇CO2 is the respiratory quotient (RQ), which provides insight into the balance of macronutrient oxidation by indicating the relative use of carbohydrates, fats, and proteins as energy sources [6].

Since IC is an indirect method based on the concentration of specific gases, therapies that alter their concentration in the blood or affect acid-base balance, such as renal replacement therapy (RRT), extracorporeal membrane oxygenation (ECMO), and liver support therapies such as the molecular adsorbent recirculating system (MARS), can potentially reduce the reliability of the obtained results. This necessitates a careful interpretation of EE measured by IC [5]. These issues have been discussed in more detail in another author’s publication [7]. 

Jonckheer et al. conducted studies assessing the removal of CO_2_ and O_2_ during continuous renal replacement therapy (CRRT) in the modality of continuous veno-venous hemofiltration (CVVH) and its impact on the measurement of REE by the IC method. These studies demonstrated that during CVVH, a fraction of CO₂ is removed, as evidenced by a significant decrease in the flow of CO₂ (QCO₂) after blood passes through the filter, while the flow of O₂ (QO₂) remains unaffected [8]. It was also shown that the changes in REE caused by CO_2_ removal during CVVH are not clinically significant. Therefore, a correction factor for EE is not required for CVVH [9]. The impact of glucose, citrate, and lactate in CRRT solutions on the energy balance was also highlighted [10]. 

Apart from CVVH, CRRT can be conducted as continuous veno-venous hemodialysis (CVVHD) or continuous veno-venous hemodiafiltration (CVVHDF). While CVVH is a method based on convection, these modalities utilize diffusion, requiring a dialysate solution. Blood flows on one side of a semipermeable membrane, while dialysate flows on the other side in the opposite direction, and solutes move across the membrane down their concentration gradients [11]. 

This study aims to evaluate the removal of O_2_ and CO_2_ during CVVHDF, a method with a dialytic component, and assess its impact on measuring REE using IC.

## Materials and methods

### Study design and patient selection

This prospective, observational, single-center study was conducted in 2021–2024 in the 1st Clinic of Intensive Therapy of the Medical University of Lublin, Poland, in accordance with the Intensive Care Unit (ICU) protocol. The study was conducted following the STROBE guidelines [12]. The inclusion criteria for the study were age over 18 years, diagnosis of sepsis (according to Sepsis-3), CRRT in CVVHDF modality, and mechanical ventilation. The exclusion criterion were pregnancy, factors affecting the reliability of indirect calorimetry (FiO_2_ > 60%, positive end-expiratory pressure (PEEP) > 12 cm H_2_O, chest drainage, presence of gases other than O_2_, CO_2_, and N_2_ in the breathing mixture), severe hemodynamic or ventilator instability, absence of informed consent.

### CRRT system

CRRT was conducted using the PrisMax^®^ (Baxter International Inc.^1^) system available in the ICU where the study was carried out. CRRT was performed using the standard Prismaflex ST150^®^ set or the oXiris^®^ set dedicated to blood purification. CRRT fluids such as predilution citrate solution (Regiocit^®^), dialysate (Biphozyl^®^), and postdilution solution (Prismasol^®^) were used. The attending physician decided on a patient’s qualification for CRRT treatment, and the choice of configuration and filter based on the patient’s clinical condition and was not part of the study. Patients in the study group were included after the initiation of CRRT treatment. In this study, treatments set on the PrisMax^®^ device as CVVHD were classified as CVVHDF due to the consistent use of citrate predilution across all CRRT modalities on this system. This classification reflects the theoretical definition of CVVHDF, which involves both convection and diffusion mechanisms, even in the absence of postdilution replacement fluid.

### Data and sample collection

Primary statistical data of the patients, including age, sex, race, and height, were obtained from medical records. On the day of the study, body weight was recorded using the bed’s built-in scale. Information regarding CRRT settings was read from the CRRT device screen. Each time, the type of filter used was recorded. Blood samples for the study were collected aseptically by puncturing the sampling port (SP) with a G25 needle attached to a collection tube (Blood Gas, S-Monovette^®^).

**Fig. 1:**
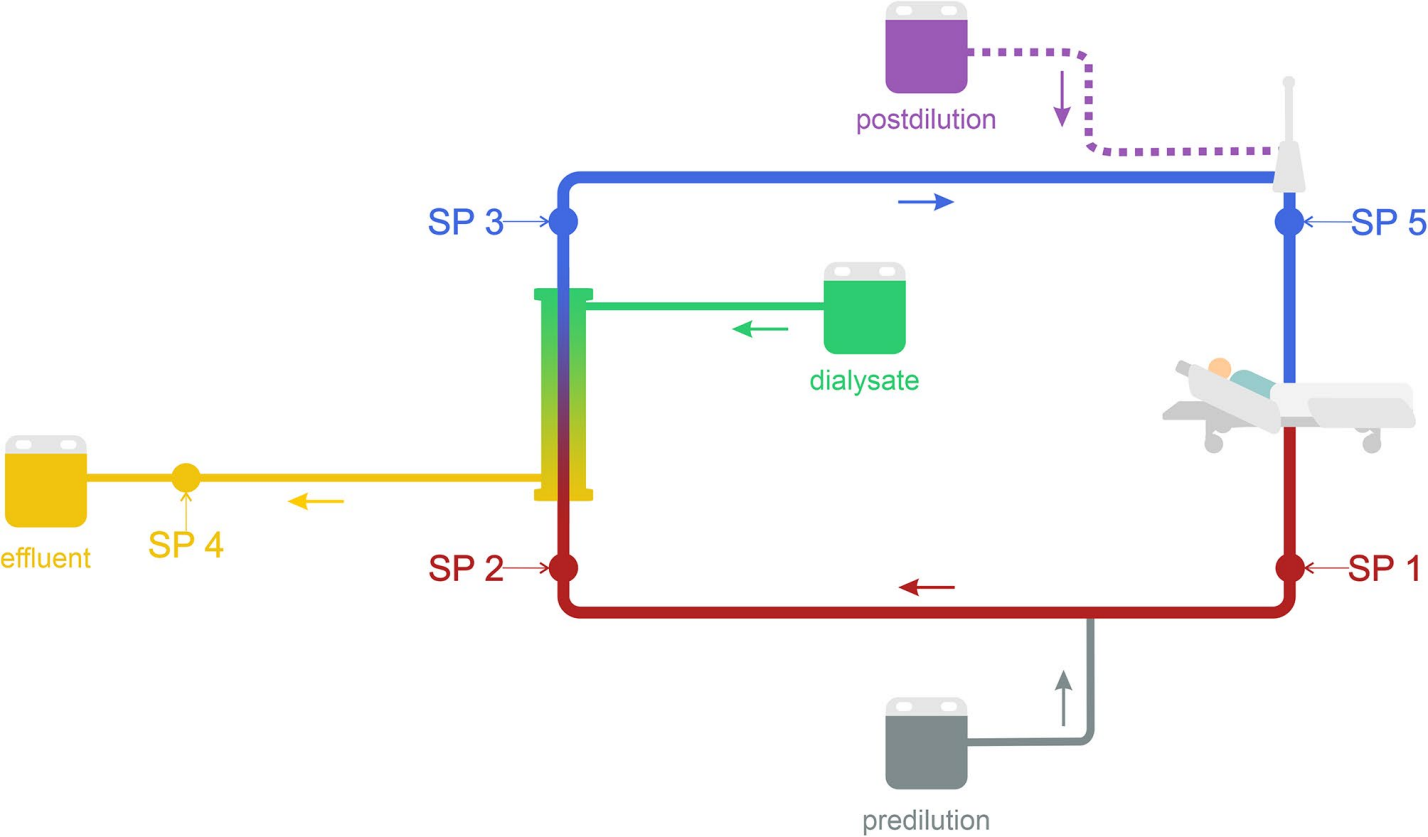
Schematic diagram of the extracorporeal circuit used for continuous veno-venous hemodiafiltration (CVVHDF) with sampling points (SPs) marked. Postdilution, indicated by a dashed line, was applied in 26 out of 31 patients

SPs were located at the distal end of the drainage lumen of the dialysis catheter (SP1), between the citrate predilution infusion port and the filter (SP2), directly after the filter (SP3), at the effluent conduit (SP4), and proximal to the return lumen of the dialysis catheter (SP5) (Fig. 1).In each collected sample, pH, bicarbonate (HCO₃⁻), total carbon dioxide (tCO₂), partial pressure of carbon dioxide (pCO₂), partial pressure of oxygen (pO₂), and hemoglobin (Hb), and Hb saturation were measured using the GEM^®^ Premier 5000 whole blood testing system.

### Indirect calorimetry

REE was measured using the COSMED Quark RMR (COSMED, Rome, Italy) calorimeter, following the *Checkpoints for successful indirect calorimetry proposed* by Oshima et al. [5] The minimum measurement duration was set at 30 min, and blood samples from the SPs were taken after at least 15 min of measurement. The measurement provided the following parameters: REE [kcal/day], V̇O_2_ [ml/min], and V̇CO_2_ [ml/min].

### Calculations

Calculations were conducted following the studies by Jonckheer et al. concerning CVVH [8, 9], with adjustments corresponding to the CRRT modalities evaluated in this study. Total CO_2_ content was calculated by the GEM^®^ Premier 5000 whole blood testing system. Total O_2_ content (tO2) was calculated as:

$${\rm{t}}{{\rm{O}}_{\rm{2}}}\,{\rm{ = }}\,{\rm{1}}{\rm{.34 \times }}\left[ {{\rm{Hb}}} \right]{\rm{ \times }}\left( {{\rm{Hb}}\;{\rm{ saturation/100}}} \right)\,{\rm{ + }}\,{\rm{0}}{\rm{.003 \times p}}{{\rm{O}}_{\rm{2}}}$$

Where: Hb – concentration of hemoglobin [g/dl], Hb saturation – percentage of hemoglobin saturation with oxygen [%], pO_2_ – partial pressure of oxygen [mmHg], tO_2_ – total oxygen content in blood [ml/dl].

Due to variable flow rates in the extracorporeal CRRT circuit, CO_2_ (QCO_2_) and O_2_ flow (QO_2_) at the specific SP were calculated by multiplying the fluid flow rate (Q) with tCO_2_ and tO_2_, respectively. The flow rate in individual SPs was calculated according to the following equations.

$$\begin{array}{l} Q_{\text{SP1}} = Q_{\text{B}} \\ Q_{\text{SP2}} = Q_{\text{B}} + Q_{\text{PRE}} \\ Q_{\text{SP3}} = Q_{\text{B}} - Q_{\text{PFR}} - Q_{\text{POST}}^* \\ Q_{\text{SP4}} = Q_{\text{PFR}} + Q_{\text{D}} + Q_{\text{PRE}} + Q_{\text{POST}}^* \\ Q_{\text{SP5}} = Q_{\text{B}} - Q_{\text{PFR}}\end{array}$$

Where: B – blood; D – dialysate; PFR – patient fluid removal; POST – postdilution (replacement fluid); PRE – predilution (pre-blood pump fluid); Q – flow rate; SP – sampling point. *In the case of CVVHDF without postdilution, the Q_POST_ value was 0.

The calculated QCO_2_ and QO_2_ were adjusted from mmol to ml using the Clapeyron equation:

$${\text{pV}}\,{\text{=}}\,{\text{nRT}}$$

Where: p - pressure of the gas [Pa], V - volume of the gas [m^3^], n - amount of substance of gas [mol], R - universal gas constant [J × mol^− 1^ × K^− 1^], T - absolute temperature of the gas [K].

The ambient temperature during the measurement was recorded. The atmospheric pressure value for Lublin on the measurement day was obtained from the data provided by the Institute of Meteorology and Water Management – National Research Institute.

The tCO_2_ of the postdilution replacement fluid was 32 mmol/l, and the dialysate fluid was 22 mmol/l (data based on the Summary of Product Characteristics). These values were converted to ml/l using the Clapeyron equation and then multiplied by the fluid flow rate to obtain values in ml/min.

Transmembrane QCO_2_ was calculated by subtracting QCO_2_ at SP3 from QCO_2_ at SP2.

$$Q_{\text{SP1}} = Q_{\text{B}}$$$$\text{Transmembrane } Q_{\text{CO}_2} = Q_{\text{CO}_2}(\text{SP2}) - Q_{\text{CO}_2}(\text{SP3})$$

When postdilution replacement fluid was used, the expected QCO_2_ at SP5 was calculated by adding the calculated QCO_2_ of the postdilution replacement fluid to the QCO_2_ at SP3.

$${\text{Expected QC}}{{\text{O}}_{\text{2}}}_{{{\text{(SP5)}}}}\,{\text{=}}\,{\text{QC}}{{\text{O}}_{\text{2}}}_{{{\text{(SP3)}}}}\,{\text{+}}\,{\text{QC}}{{\text{O}}_{\text{2}}}_{{{\text{(POST)}}}}$$

The V̇CO_2_ balance for CRRT was calculated by subtracting the QCO_2_ from the dialysate and the postdilution replacement fluid from the QCO_2_ in the effluent.

$${\rm{\dot V C}}{{\rm{O}}_{\rm{2}}}_{{\rm{(CRRT)}}}\,{\rm{ = }}\,{\rm{QC}}{{\rm{O}}_{\rm{2}}}_{{\rm{(SP4)}}}{\rm{ - QC}}{{\rm{O}}_{\rm{2}}}_{{\rm{(D)}}}{\rm{ - QC}}{{\rm{O}}_{\rm{2}}}_{{\rm{(POST)}}}$$

Since the COSMED Quark ICU calorimeter calculates REE based on the abbreviated Weir equation, to obtain consistent results, the V̇O₂ and V̇CO₂ values from the calorimeter measurements were extracted and then applied to the full Weir equation [13, 14].

$${\rm{Abbreviated}}\,{\rm{ Weir}}\,{\rm{ equation: REE = (\dot V }}{{\rm{O}}_{\rm{2}}}{\rm{ \times 3}}{\rm{.9}}\,{\rm{ + }}\,{\rm{\dot V C}}{{\rm{O}}_{\rm{2}}}{\rm{ \times 1}}{\rm{.1) \times 1440}}$$

$${\rm{Full}}\,{\rm{ Weir}}\,{\rm{ equation: REE = (\dot V }}{{\rm{O}}_{\rm{2}}}{\rm{ \times 3}}{\rm{.941}}\,{\rm{ + }}\,{\rm{\dot V C}}{{\rm{O}}_{\rm{2}}}{\rm{ \times 1}}{\rm{.11) \times 1440}}$$

Where: REE – resting energy expenditure [kcal/day], V̇*CO*_2_ – carbon dioxide production [l/min], V̇*O*_2_ – oxygen consumption [l/min].

The true V̇CO_2_ was defined, according to Jonckheer et al. [9], as the actual CO_2_ production of the body by adding the CO_2_ that is removed or added during the CRRT process into the equation. The true V̇CO_2_ was calculated by adding V̇CO_2_ measured by indirect calorimetry and QCO_2_ from effluent measured in SP4, minus QCO_2_ from the dialysate, by subtracting QCO_2_ from the postdilution replacement fluid (if applicable). The true REE was calculated by including this true V̇CO_2_ in the Weir equation.

$$\eqalign{& {\rm{True}}\,{\rm{ \dot V C}}{{\rm{O}}_{\rm{2}}}{\rm{ = \dot V C}}{{\rm{O}}_{\rm{2}}}_{{\rm{(IC)}}}\,{\rm{ + }}\,{\rm{\dot V C}}{{\rm{O}}_{\rm{2}}}_{{\rm{(CRRT)}}} \cr & {\rm{True}}\,{\rm{ REE = (\dot V }}{{\rm{O}}_{\rm{2}}}_{\left( {{\rm{IC}}} \right)}{\rm{ \times 3}}{\rm{.941}}\,{\rm{ + }}\,{\rm{True}}\,{\rm{ \dot V C}}{{\rm{O}}_{\rm{2}}}_{\left( {{\rm{IC + CRRT}}} \right)}{\rm{ \times 1}}{\rm{.11) \times 1440}} \cr}$$

The obtained results were compared with the estimated EE calculated using the Harris-Benedict equation [15]:

$$\eqalign{{\rm{For}}\,{\rm{ men: REE}}\,{\rm{ = }}\,{\rm{66}}{\rm{.473 }} & {\rm{ + }}\left( {{\rm{13}}{\rm{.7516 \times M}}} \right){\rm{ }} \cr & {\rm{ + }}\left( {{\rm{5}}{\rm{.0033 \times H}}} \right){\rm{ - }}\left( {{\rm{6}}{\rm{.755 \times A}}} \right) \cr}$$

$$\eqalign{{\rm{For}}\,{\rm{ women: REE}}\,{\rm{ = }}\,{\rm{655}}{\rm{.0955 }} & {\rm{ + }}\left( {{\rm{9}}{\rm{.5634 \times M}}} \right){\rm{ }} \cr & {\rm{ + }}\left( {{\rm{1}}{\rm{.8496 \times H}}} \right){\rm{ - }}\left( {{\rm{4}}{\rm{.6756 \times A}}} \right) \cr}$$

Where: A – age [years], H – height [cm], M – body mass [kg], REE – resting energy expenditure [kcal/day].

The respiratory quotient (RQ) was calculated by dividing the rate of CO₂ production by the rate of O₂ consumption; for the measured RQ, data from IC were used, while for the true RQ, VO₂ data from IC and true VCO₂ were applied.

$$\eqalign{& {\rm{Measured}}\,{\rm{ RQ}}\,{\rm{ = }}\,{\rm{\dot V C}}{{\rm{O}}_{\rm{2}}}_{{\rm{(IC)}}}{\rm{/ \dot V }}{{\rm{O}}_{\rm{2}}}_{{\rm{(IC)}}} \cr & {\rm{True}}\,{\rm{ RQ}}\,{\rm{ = }}\,{\rm{True}}\,{\rm{\dot V C}}{{\rm{O}}_{\rm{2}}}_{{\rm{(IC + CRRT)}}}{\rm{/ \dot V }}{{\rm{O}}_{\rm{2}}}_{{\rm{(IC)}}} \cr}$$

### Statistical analysis

Data were analyzed using Statistica 13 software (StatSoft). Data expressed on a quantitative scale were presented as mean with standard deviation (SD); for selected variables, median and range were also provided. Data expressed on a qualitative scale were presented as the number and percentage of the sample. The Spearman correlation analysis was used to evaluate the relationship between the V̇CO₂ balance for CRRT and parameters describing CO₂ content at SP1. Student’s t-test for dependent samples and Friedman test were used to assess how the parameters under study changed before and after the filter. All statistical tests were two-tailed, and a p-value of less than 0.05 was considered statistically significant.

### Ethical issues

All research procedures complied with the Declaration of Helsinki and the Medical University of Lublin Bioethics Committee guidelines. Approval was obtained from the Medical University of Lublin Bioethics Committee, approval number KE-0254/74/2021. Informed consent was obtained from each patient to participate in the study. If the patient’s condition did not permit this, consent was obtained from the patient’s legally authorized representative or closest family member.

## Results

A total of 31 patients participated in the study, and a single measurement was performed for each patient. Table 1. summarizes patient characteristics.

**Table 1:** Patient characteristics. Data are presented as mean ± standard deviation (SD)

	*n* = 31
Age [years]	70.32 ± 8.56
Sex	
Female	11 (35.48%)
Male	20 (64.52%)
Race	
Caucasian	31 (100.00%)
BMI [kg/m^2^]	31.11 ± 4.80
APACHE II^a^	13.90 ± 4.78
APS^a^	8.65 ± 4.48
SOFA^a^	11.42 ± 2.22
Renal replacement therapy parameters	
Configuration of CVVHDF	
Predilution	31 (100.00%)
Postdilution	26 (83.87%)
Flow rates	
Q_B_ [ml/h]	7939.36 ± 1141.41
Q_PFR_ [ml/h]	120.16 ± 80.17
Q_D_ [ml/h]	1511.29 ± 401.40
Q_PRE_ [ml/h]	1307.19 ± 199.86
Q_POST_ [ml/h]	417.74 ± 228.24

^a^ The scores in the SOFA, APS, and APACHE II scales were calculated, excluding the assessment of central nervous system function (Glasgow Coma Scale) due to patient sedation

**Abbreviations**: APACHE II - Acute Physiology and Chronic Health Evaluation II score, APS - Acute Physiology Score, BMI - body mass index, CVVHDF - continuous veno-venous hemodiafiltration, Q_B_ - blood flow rate, Q_D_ - dialysate flow rate, Q_PFR_ - patient fluid removal rate, Q_POST_ - postdilution (replacement fluid) flow rate, Q_PRE_ - predilution (pre-blood pump fluid) flow rate, SOFA - Sequential Organ Failure Assessment score

### Comparison of QCO₂

**Fig. 2:**
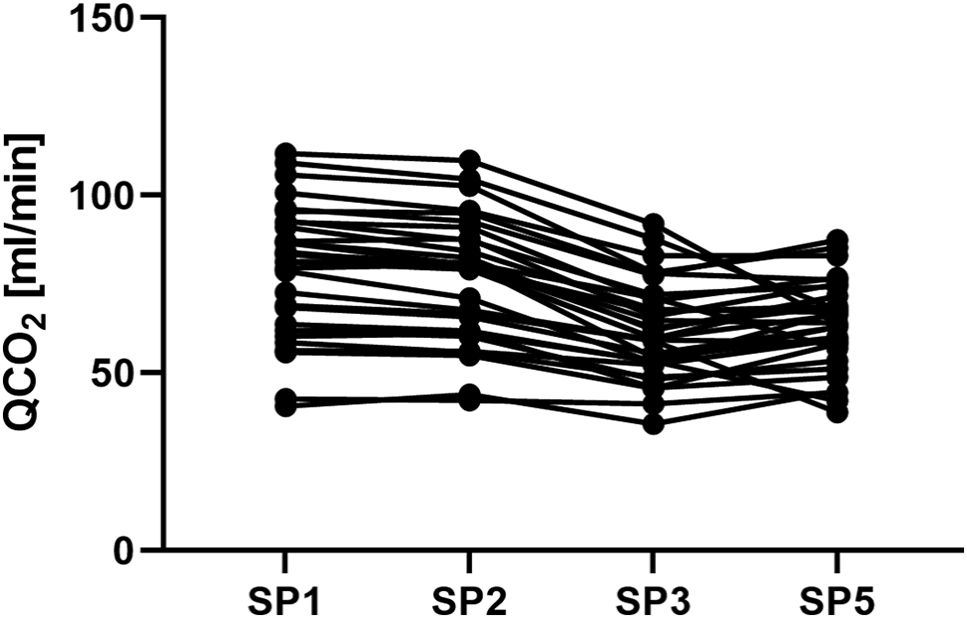
Evolution of QCO₂ in the extracorporeal CRRT circuit. SP4 was omitted as it is not situated in the extracorporeal blood circuit and is not suited to represent the evolution of QCO₂ in the blood. The CQO₂ measured before the filter (SP1 and SP2) was statistically significantly higher than those measured after the filter (SP3 and SP5). Abbreviations: SP – sampling point, QCO₂ – carbon dioxide flow

The QCO₂ measured before the filter (SP1 and SP2) was statistically significantly higher than those measured after the filter (SP3 and SP5) (Fig. 2). The mean QCO₂ values, along with medians and ranges for each sampling point (SP) are presented in Table 2. The QCO₂ decreased significantly between SP2 and SP3 (*p* < 0.0001).

**Table 2:** QCO₂ and QO₂ at sampling points (SPs). Data are presented as mean ± standard deviation (SD) and median (range)

Sampling point	QCO₂ [ml/min]		QO₂ [ml/min]	
	Mean ± SD	Median (range)	Mean ± SD	Median (range)
SP1	78.72 ± 18.43	81.38 (40.72–111.78)	10.30 ± 3.88	9.89 (2.99–18.96)
SP2	76.26 ± 17.33	79.73 (42.30–109.84)	10.47 ± 4.19	10.45 (2.58–19.03)
SP3	62.12 ± 13.64	59.86 (35.67–91.89)	10.58 ± 3.98	10.22 (2.62–19.02)
SP4	28.69 ± 6.34	29.88 (14.64–43.74)	N/A ^a^	N/A ^a^
SP5	63.83 ± 12.41	64.02 (39.00–87.40)	10.82 ± 3.96	10.35 (3.33–19.67)

^a^The effluent does not contain hemoglobin; therefore, QO₂ was not assessed at SP4

Abbreviations: SP – sampling point, QCO₂ – carbon dioxide flow, QO₂ – oxygen flow

**Fig. 3:**
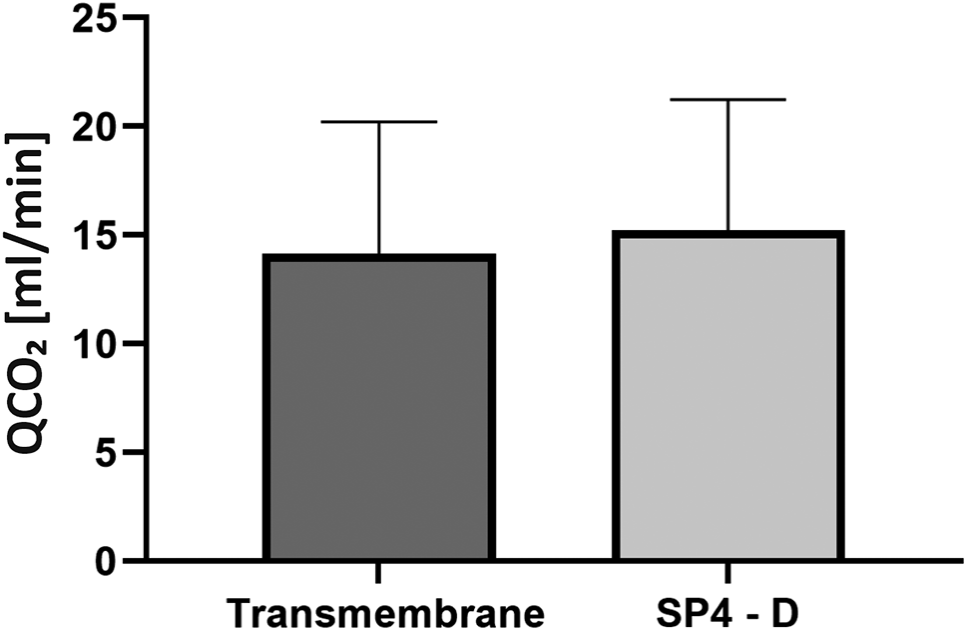
Comparison of mean transmembrane QCO₂ (14.14 ± 6.09 ml/min) and QCO₂ measured in the effluent, adjusted by subtracting the QCO₂ calculated for the dialysate (15.23 ± 6.01 ml/min). A paired t-test for dependent samples demonstrated that the difference between the transmembrane QCO₂ and the adjusted QCO₂ measured in the effluent is not significant (*p* = 0.063)

The difference between the transmembrane QCO₂ (14.14 ± 6.09 ml/min) and the QCO₂ measured in the effluent (SP4) (15.23 ± 6.01 ml/min) is not significant (*p* = 0.063) (Fig. 3). In this comparison, the QCO₂ measured in the effluent (SP4) was adjusted by subtracting the calculated QCO₂ addition due to the dialysate used over the filter.

**Fig. 4:**
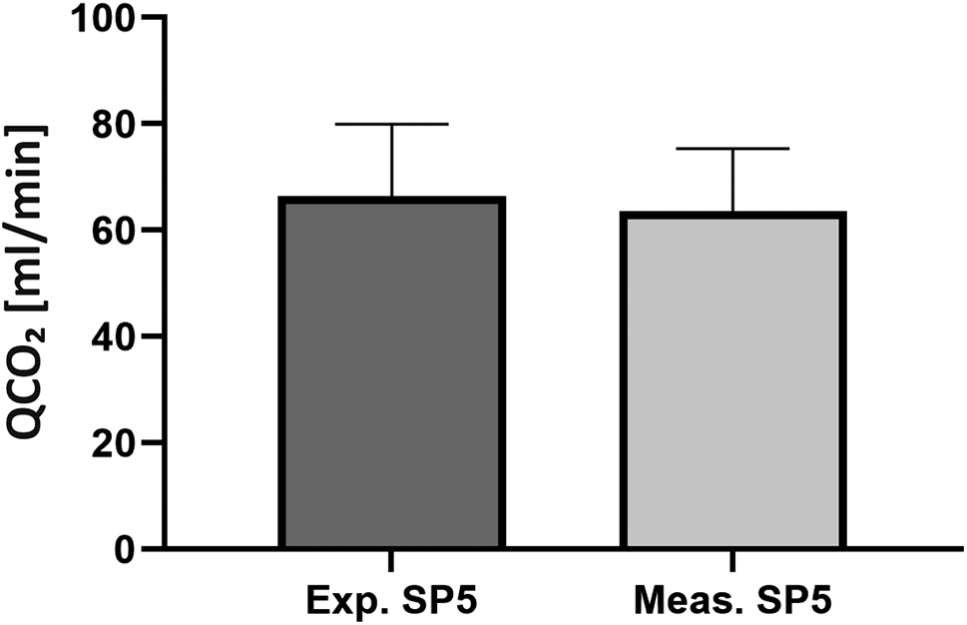
Comparison of expected QCO₂ at SP5 (66.39 ± 13.53 ml/min), calculated by adding the calculated QCO₂ of the postdilution fluid to the QCO₂ at SP3 and measured QCO₂ at SP5 (63.61 ± 11.72 ml/min) when postdilution replacement fluid was used (*n* = 26). A paired t-test for dependent samples demonstrated that the difference between the expected and measured QCO₂ at SP5 is not significant (*p* = 0.156)

When a postdilution replacement fluid was used (*n* = 26), the expected QCO₂ at SP5 was calculated by adding the calculated QCO₂ of the postdilution fluid to the QCO₂ at SP3 and compared to the measured QCO₂ at SP5. Expected QCO₂ at SP5 averaged 66.39 ± 13.53 ml/min, while the measured QCO₂ averaged 63.61 ± 11.72 ml/min (Fig. 4). No significant difference was found between the expected and measured QCO₂ at SP5 (*p* = 0.156).

The V̇CO₂ balance for CRRT was calculated by subtracting the QCO₂ from the dialysate and the postdilution replacement fluid from the QCO₂ in the effluent and averaged 9.832 ± 5.827 ml/min. A strong and statistically significant correlation was demonstrated between the V̇CO₂ balance for CRRT and both QCO₂ and tCO₂ at SP1. Specifically, the Spearman’s rank correlation coefficient was 0.885 for QCO₂ SP1 (*p* < 0.0001) and 0.880 for tCO₂ SP1 (*p* < 0.0001).

### Comparison of QO₂

**Fig. 5:**
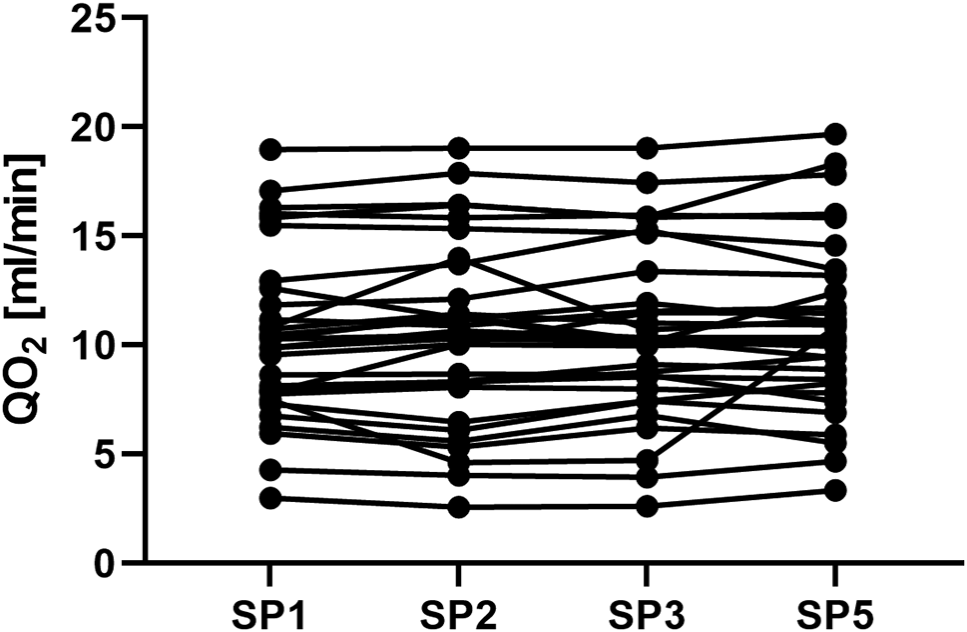
Evolution of QO₂ in the extracorporeal CRRT circuit. The effluent does not contain hemoglobin; therefore, QO₂ was not assessed at SP4. No statistically significant difference was found between QO2 at the SPs. Abbreviations: SP – sampling point, QO₂ – oxygen flow

The mean QO₂ values, along with medians and ranges for each SP are presented in Table 2. No statistically significant difference was found between QO₂ at SP1 and SP5 (Fig. 5). The effluent does not contain hemoglobin; therefore, QO₂ was not assessed at SP4.

### Resting energy expenditure

**Fig. 6:**
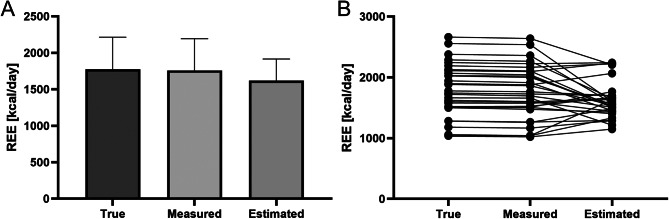
(**A**) Comparison of mean resting energy expenditure (REE) values obtained using different methods: true REE calculated by applying true V̇CO2 in the Weir equation, measured REE obtained by using V̇CO2 and V̇O2 values measured by a calorimeter, and estimated REE calculated using the Harris-Benedict equation. (**B**) Comparison of REE values obtained using the above methods for individual measurements. The Friedman test for dependent samples found a significant difference between true REE and measured REE (*p* = 0.003) and between true REE and estimated REE (*p* = 0.048)

REE values were obtained using three methods: true REE calculated by applying true V̇CO₂ in the Weir equation, measured REE obtained by using V̇CO₂ and V̇O₂ values measured by a calorimeter, and estimated REE calculated using the Harris-Benedict equation. Table 3; Fig. 6A present the mean values of REE obtained using different methods. The difference between true REE and measured REE was, on average, 15.69 ± 9.34 kcal/day, while the difference between true REE and estimated REE was 154.93 ± 359.30 kcal/day. The Friedman test for dependent samples found a statistically significant difference between true REE and measured REE (*p* = 0.0029) and between true REE and estimated REE (*p* = 0.0475) (Fig. 6B).

**Table 3:** Average resting energy expenditure (REE) values obtained using three different methods. The data are presented as mean ± standard deviation (SD)

	REE [kcal/day]
True	1774.28 ± 438.20
Measured	1758.59 ± 434.06
Estimated (Harris-Benedict equation)	1619.36 ± 295.46

### Respiratory quotient

**Fig. 7:**
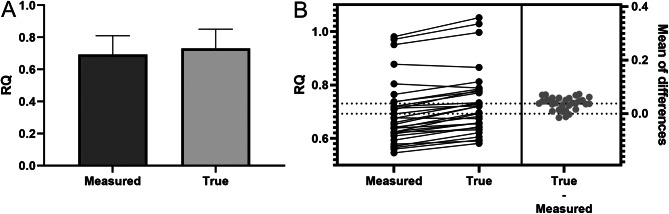
(**A**) Comparison of mean respiratory quotient (RQ) values obtained using different methods: measured RQ based solely on V̇O₂ and V̇CO₂ measured by indirect calorimetry (IC), and true RQ calculated by including true V̇CO₂. (**B**) Comparison of RQ values obtained using the above methods for individual measurements. A paired t-test for dependent samples demonstrated a significant difference between the measured RQ and true RQ (*p* < 0.0001)

The RQ was obtained in two ways: first, based solely on V̇O₂ and V̇CO_2_ measured by IC, and second, by including true V̇CO₂ (true RQ). The mean measured RQ value was 0.693 ± 0.118, while the mean true RQ value was 0.731 ± 0.121 (Fig. 7A). A paired t-test was conducted to compare the RQ values obtained from the same subjects using two different methods. The results demonstrated a statistically significant difference between the measured RQ (based on V̇O₂ and V̇CO₂ from indirect calorimetry) and the true RQ (calculated by including true V̇CO₂), with a p-value of < 0.0001 (Fig. 7B).

## Discussion

This study aimed to evaluate the effects of CVVHDF on O₂ and CO₂ removal and the measurement of REE using IC. Our findings may contribute to a better understanding of how these modalities influence gas exchange and the accuracy of REE measurements using IC in critically ill patients with sepsis.

Our results indicate that QCO₂ levels measured before the filter (SP1 and SP2) were significantly higher than those measured after the filter (SP3 and SP5). This finding is consistent with previous studies by Jonckheer et al., who demonstrated that CRRT using CVVH, a convection-based technique, reduces CO₂ levels [8]. Our study focused on CVVHDF, which combines diffusion and convection techniques. The significant decrease in QCO₂ between SP2 and SP3 indicates that CO₂ is removed during blood flow through the filter in these modalities. The similarity between transmembrane QCO₂ and QCO₂ at SP4 suggests that measurement at SP4 reliably reflects CO₂ removal without patient blood loss. Since CVVHDF utilizes both dialysate and replacement fluids, the bicarbonate ion (HCO₃⁻) content in these solutions must be accounted for when calculating the V̇CO₂ balance and its impact on REE measurements using IC.

Our study found no significant difference between the expected and measured QCO₂ at SP5. It should be noted that some CO₂ may have been removed in the deaeration chamber. Jonckheer et al. reported the removal of 1.3 ml/min of CO₂ during CVVH, which constitutes a minor amount compared to typical V̇CO₂ levels in ICU patients [8, 16]. In this study, due to the use of postdilution fluid containing HCO₃⁻, it was not possible to assess CO₂ loss in the deaeration chamber. However, the non-significant differences between the measured and expected QCO₂ at SP5 suggest that this loss may not be substantial.

The QO₂ measurements showed no significant differences between SP1 and SP5. This result aligns with the findings by Jonckheer et al., who reported that CVVH does not significantly impact V̇O₂ [8]. The absence of hemoglobin in the effluent precluded the assessment of QO₂ at SP4, but the consistent QO₂ values before and after the filter indicate stable oxygen flow and minimal interference by the CRRT process.

The comparison of REE obtained via different methods showed a significant difference between REE measured by IC and true REE, with an average discrepancy of 15.69 ± 9.34 kcal/day, less than 1% of the measured REE. This confirms the utility of IC for assessing REE in patients undergoing CVVHDF without a correction factor. In contrast, the Harris-Benedict equation showed a much larger discrepancy of 154.93 ± 359.30 kcal/day. Although CRRT can influence REE measurements, IC provides more accurate results than predictive equations and remains a preferable method for critically ill patients. A systematic review by Mtaweh et al. highlighted key factors influencing energy expenditure in critically ill patients, including minute volume, weight, age, burns, and sedation, while emphasizing the limitations of predictive equations and the need for further investigation [17]. Ponce et al. found correlations between REE and clinical parameters such as C-reactive protein, minute volume, and BMI in patients undergoing RRT [18]. Fishman and Singer, supported by ESPEN recommendations, advocate for repeated IC measurements in CRRT patients to capture dynamic changes in energy requirements [3, 19, 20]. We suggest that IC remains the best method for assessing REE in ICU patients treated with CRRT for clinical purposes. However, for research purposes, this measurement should be adjusted for the V̇CO₂ balance from CRRT. Regarding diffusion-based techniques, the equation for true REE proposed by Jonckheer et al. in the MECCIAS trial [9] should be modified by subtracting the QCO₂ derived from the HCO₃⁻ contained in the dialysate from the QCO₂ measured at SP4.

Our findings demonstrated that the mean V̇CO₂ balance for CRRT was 9.832 ± 5.827 ml/min. A strong and significant correlation was shown between the V̇CO₂ balance for CRRT and both QCO₂ and tCO₂ in the blood flowing from the patient (SP1). tCO₂ is a derivative of pCO₂ and HCO₃⁻, and it is typically reported by point-of-care blood testing systems as a calculated parameter. It measures the total amount of CO₂ in the blood, and approximately 95% of the CO₂ in the blood is present in the form of HCO₃⁻ [21]. This observation suggests that higher levels of tCO₂ in the patient’s blood may influence the outcome of CRRT as measured by IC.

RQ, defined as the ratio of CO₂ production to O₂ consumption, reflects substrate utilization. Typical values range from 1.0 (glucose oxidation) to 0.7 (fat oxidation), with deviations below 0.7 suggesting ketone body metabolism. The physiological range of RQ is 0.67–1.3, and a mixed oral diet typically produces an RQ of 0.8 [6, 22–24]. Our findings showed that the mean measured RQ value was 0.693 ± 0.118, while the mean true RQ value, calculated by including true V̇CO₂, was 0.731 ± 0.121, and a paired t-test demonstrated a significant difference between the two methods, with a p-value of < 0.0001. The paired t-test suggests CRRT may significantly impact RQ, potentially affecting substrate interpretation and the reliability of the measurement.

This single-center study with a small sample size (*n* = 31) may limit generalizability. Configuration variability based on clinical judgment could also influence results. More extensive multicenter studies are needed to confirm these results across different patient populations and clinical settings. Although IC is the gold standard for measuring REE, it has limitations, especially in patients with unstable clinical conditions or those receiving high levels of ventilatory support (FiO_2_ > 60%, PEEP > 12 cm H_2_O) [5, 7]. The exclusion criteria related to these factors may have introduced selection bias. However, since these conditions remain contraindications for performing IC, this selection bias should not have consequences in clinical practice and research. Critically ill patients, especially those with sepsis, experience rapid and significant changes in their metabolic status. The study captured measurements at specific time points, but continuous monitoring might provide a more comprehensive understanding of the metabolic changes over time. The study relied on calculating tCO₂ using the GEM^®^ Premier 5000 whole blood testing system rather than direct measurement. The equations used for these calculations may not accurately reflect the non-physiological state of blood alkalization induced by bicarbonate from the replacement fluid [8]. Future studies should aim to address these limitations by including larger sample sizes, employing multicenter designs, and using continuous monitoring techniques to capture dynamic changes in energy expenditure. Additionally, direct measurement of tCO₂ and more standardized CRRT settings would enhance the accuracy and reliability of the findings.

## Conclusion

This study demonstrated that CVVHDF effectively reduces CO₂ levels in the extracorporeal CRRT circuit, as evidenced by a significant decrease in QCO₂ between SP2 and SP3. The stable QO₂ measurements between SP1 and SP5 indicate minimal impact on oxygen levels. Although CRRT slightly altered REE measurements using IC, these differences were minor compared to discrepancies observed with predictive equations, reaffirming the utility of IC for clinical REE assessment. Larger, multicenter studies are needed to confirm these findings across diverse patient populations and clinical settings. Future research should include direct measurement of tCO₂ to improve V̇CO₂ calculations and the assessment of CO₂ loss in the deaeration chamber.

## Data Availability

The datasets used and analysed during the current study are available from the corresponding author on reasonable request.

## Footnotes

1 Baxter, oXiris, Prismaflex, PrisMax, Regiocit, Biphozyl, Prismasol are trademarks of Baxter International Inc. or its subsidiaries.
